# Genome-wide protein QTL mapping identifies human plasma kallikrein as a post-translational regulator of serum uPAR levels

**DOI:** 10.1096/fj.13-240879

**Published:** 2014-02

**Authors:** Michael A. Portelli, Mateusz Siedlinski, Ceri E. Stewart, Dirkje S. Postma, Maartje A. Nieuwenhuis, Judith M. Vonk, Peter Nurnberg, Janine Altmuller, Miriam F. Moffatt, Andrew J. Wardlaw, Stuart G. Parker, Martin J. Connolly, Gerard H. Koppelman, Ian Sayers

**Affiliations:** * Division of Respiratory Medicine, University of Nottingham, Queen's Medical Centre, Nottingham, UK;; † Department of Epidemiology and; ‡ Department of Pulmonology, and; § Department of Paediatric Pulmonology and Paediatric Allergology, Beatrix Children's Hospital, Groningen Research Institute for Asthma and COPD (GRIAC), University of Groningen, University Medical Center Groningen, Groningen, The Netherlands;; ‖ Cologne Center for Genomics, University of Cologne, Cologne, Germany;; ¶ National Heart and Lung Institute, Imperial College London, London, UK;; # Institute for Lung Health, Immunity, and Inflammation, University of Leicester, Leicester, UK;; ** University of Sheffield, Sheffield, UK; and; †† Freemasons Department of Geriatric Medicine, University of Auckland, Auckland, New Zealand

**Keywords:** GWAS, proteolysis, respiratory disease, HBECs, cellular proliferation and wound repair

## Abstract

The soluble cleaved urokinase plasminogen activator receptor (scuPAR) is a circulating protein detected in multiple diseases, including various cancers, cardiovascular disease, and kidney disease, where elevated levels of scuPAR have been associated with worsening prognosis and increased disease aggressiveness. We aimed to identify novel genetic and biomolecular mechanisms regulating scuPAR levels. Elevated serum scuPAR levels were identified in asthma (*n*=514) and chronic obstructive pulmonary disease (COPD; *n*=219) cohorts when compared to controls (*n*=96). In these cohorts, a genome-wide association study of serum scuPAR levels identified a human plasma kallikrein gene (*KLKB1*) promoter polymorphism (rs4253238) associated with serum scuPAR levels in a control/asthma population (*P*=1.17×10^−7^), which was also observed in a COPD population (combined *P*=5.04×10^−12^). Using a fluorescent assay, we demonstrated that serum *KLKB1* enzymatic activity was driven by rs4253238 and is inverse to scuPAR levels. Biochemical analysis identified that *KLKB1* cleaves scuPAR and negates scuPAR's effects on primary human bronchial epithelial cells (HBECs) *in vitro*. Chymotrypsin was used as a proproteolytic control, while basal HBECs were used as a control to define scuPAR-driven effects. In summary, we reveal a novel post-translational regulatory mechanism for scuPAR using a hypothesis-free approach with implications for multiple human diseases.—Portelli, M. A., Siedlinski, M., Stewart, C. E., Postma, D. S., Nieuwenhuis, M. A., Vonk, J. M., Nurnberg, P., Altmuller, J., Moffatt, M. F., Wardlaw, A. J., Parker, S. G., Connolly, M. J., Koppelman, G. H., Sayers, I. Genome-wide protein QTL mapping identifies human plasma kallikrein as a post-translational regulator of serum uPAR levels.

The urokinase plasminogen activator receptor (uPAR; also known as PLAUR and CD87) is a cellular receptor for urokinase (uPA), which activates the plasminogen/plasmin activation cycle and its extracellular proteolytic cascade (1). uPAR also acts through nonproteolytic functions, where associations with a number of cell-bound factors, such as integrins and G-protein-coupled receptors, allow uPAR to stimulate signaling cascades, regulating functions such as cytoskeletal dynamics, cellular adhesion, and cellular migration (2). Through its proteolytic and nonproteolytic cascades, uPAR is involved in extracellular matrix remodeling, cell migration, coagulation, cellular proliferation, release of inflammatory cytokines, and growth factor activation (1).

uPAR exists as a glycosylphosphatidylinositol (GPI)-anchored membrane protein [commonly defined as membrane uPAR (muPAR); refs. 3–5] translated from a 1.4-kb mRNA (4) from the *PLAUR* gene on the 19q13 chromosome (5). The *PLAUR* gene consists of 7 exons (6), exon 1 containing the 5′ untranslated region (5′UTR) and a signal peptide, while exons 2–3, 4–5, and 6–7 encode globular domains D_I_, D_II_, and D_III_, respectively (as numbered from the N terminus; ref. 6), where D_I_ and D_II_ are known to be involved in uPA binding and chemotaxis, and D_III_ is important for integrin binding (*e.g.*, α5β1; ref. 6).

This membrane-bound receptor is ubiquitously expressed on multiple cell types and tissues (6) and is implicated in a number of diseases, including asthma (7, 8), cancer (9, 10), epilepsy (11), and cardiovascular disease (12), suggesting an important biological role for this protein. This is further supported at the cellular level, where uPAR has been reported to be involved in efficient epithelial wound repair (8), neutrophil recruitment during cellular inflammation, tumor invasiveness, and metastasis (13, 14), especially in breast cancer (15).

Apart from the membrane-bound form of the receptor, a soluble cleaved form of uPAR (scuPAR) is also known to exist. This arises from glycolytic and lipolytic cleavage of muPAR's GPI anchor, *via* a number of molecules, such as GPI-specific phospholipase-D and cathepsin G (16). The resulting scuPAR is known to exist under normal physiological conditions in several biological fluids, including serum (17) and induced sputum (18). Furthermore, scuPAR has a role in trafficking of neutrophils to sites of inflammation (2) and local mobilization of stem cells (19). While total scuPAR levels are stable in healthy individuals (4), high circulating levels of the soluble receptor have been described in a number of diseases, including ventilator associated pneumonia (20), HIV (21), cardiovascular disease, and cancer (22). In these disease states, scuPAR is frequently reported to act as a biomarker, with high scuPAR levels linked to patient mortality rates and increased disease aggressiveness (22, 23). This has previously led to the suggestion that scuPAR may be of value as a prognostic marker of disease. However, recent investigations have identified a direct role for elevated levels of freely circulating scuPAR in the serum of subjects with focal segmental glomerulosclerosis (FSGS; ref. 24). Here, scuPAR did not only act as a marker of disease, but also played an active role in the development and modulation of the disease through interaction with β_3_ integrins on the kidney's visceral epithelial cells (podocytes; ref. 24). This functional role for scuPAR is independent of muPAR function and beyond that of a uPA decoy receptor (8). This therefore indicates that changes in freely circulating scuPAR may be important to the development and modulation of multiple human diseases. Therefore, furthering our understanding of the mechanism regulating serum scuPAR levels would be of importance in understanding the biology underlying the receptor's role in multiple disease states.

A number of studies have suggested that uPAR may play a role in obstructive lung disease. We have previously identified *PLAUR* as an asthma susceptibility gene (7). Here single-nucleotide polymorphisms (SNPs) spanning the gene and its untranslated regions were found to be associated with asthma susceptibility, bronchial hyperresponsiveness, lung function decline, and, importantly, with increased serum scuPAR levels (7). Similarly, in further independent studies, we have shown that *PLAUR* SNPs are associated with baseline lung function in smokers (25) and that uPAR is elevated in the airway epithelium in asthma (8).

This study aimed to determine whether scuPAR is elevated in patients with obstructive lung disease and to identify a novel regulatory mechanism for scuPAR, through a genome-wide association study (GWAS), which may be of relevance to scuPAR associated disease. Here we demonstrate that serum scuPAR is significantly elevated in asthma and COPD patient serum. Following this we successfully employed a GWAS, identifying a novel genetic mechanism determining uPAR levels and successfully demonstrated that the biochemical and functional basis of the described association is driven by a human plasma kallikrein gene (*KLKB1*) cleavage of uPAR. Finally we utilized *in vitro* epithelial cell models to identify that the effects of scuPAR on primary human cell function are negated in the presence of KLKB1, further confirming the important modulating role of KLKB1 on uPAR function.

## MATERIALS AND METHODS

### Cohort characteristics

Asthma (*n*=514) and control (*n*=104) subjects were selected from 200 families and 407 trios, both ascertained through an asthmatic proband and characterized using a standardized study protocol, with asthma from the northern part of the Netherlands and studies with an identical protocol, as described previously (26). Selection was based on whether subjects had serum available for this study. Ethical approval was obtained from the University of Groningen Medical Ethics Committee (MEC 96/04/077 and MEC 90/09/178).

COPD (*n*=219) subjects were recruited from UK centers based on physician- and spirometry-defined COPD (FEV_1_/FVC<70%; FEV_1_<80%), Caucasian, >40 yr old, smokers with >10 pack-yr history. Subjects with a previous diagnosis of asthma were excluded (25). Ethical approval was obtained from the Multicenter Research Ethics Committee (MREC/99/4/001), and informed consent from all subjects was obtained.

### Analysis of soluble protein levels

Levels of scuPAR in human serum were determined using a Duoset ELISA (R&D Systems, Abingdon, UK). Samples were diluted 1:8 in 1% BSA in PBS. Levels of active supernatant plasmin were determined in undiluted serum using the SensoLyte AFC plasmin activity assay kit (Cambridge Bioscience, Cambridge, UK). Assays were read at 450 nm (background subtraction 570 nm), using a Flexstation 3 microplate reader (Molecular Devices, Wokingham, UK).

### GWAS

A GWAS involving a data set of 295,196 SNPs genotyped on the Illumina 300 and 370 chips (Illumina, Inc., San Diego, CA, USA) was carried out in 104 control subjects and 480 subjects with doctor-diagnosed asthma and hyperresponsiveness (histamine PC_20_<32 mg/ml) with log_10_ serum scuPAR levels as the outcome. Quality control included a SNP call rate of 0.95, and the SNPs were in Hardy Weinberg equilibrium (*P*>1×10^−7^). Related subjects were excluded using a PI HAT cutoff of 0.1875, and ethnic outliers and cryptically related subjects were removed with smartpca 8000 software (27). Associations were investigated using linear regression in PLINK 1.07 (additive model; ref. 28) with adjustment taken into consideration for 2 eigenvectors in cases. Closer analyses of regions determined to be of interest were carried out using LocusZoom 1.1 (29). A value of *P* = 1.69 × 10^−7^ was considered significant using a Bonferroni correction. Inflation factors λ were calculated using WGAViewer 1.26 software (30), and QQ and Manhattan plots were generated using R 2.15.0 (31). Meta-analysis was a fixed effect analysis carried out in PLINK (28).

### mRNA by SNP browser

Associations between changes in *KLKB1* mRNA levels and *PLAUR* expression were investigated using the mRNA by SNP browser (32). This software incorporates a generic eQTL database and provides a graphic interface for browsing association between 54,675 mRNA transcript levels and 406,912 SNPs. For each transcript, the browser provides association test statistics (*P*<0.001), estimates of effect size and allele information across the genome. Data were obtained from Epstein-Barr virus-transformed lymphoblastoid cell lines taken from 206 families of British descent selected through a proband of childhood asthma, with cells taken from affected subjects and their sibling pairs regardless of whether the sibling pair had asthma (*n*=397 sibling pairs; 11 half sibling pairs; ref. 32).

### Prediction of changes in transcription factor binding sites

The region containing the SNP rs4253238 ranging from the *KLKB1* gene transcriptional start site up to 100 bp upstream of the SNP was run through 3 transcription factor prediction databases: MatInspector TF Mining tool (Genomatix Software GmbH, Munich, Germany; http://www.genomatix.de); Transcription Element Search System (TESS; Computational Biology and Informatics Laboratory, University of Pennsylvania, Philadelphia, PA; http://www.cbil.upenn.edu/cgi-bin/tess/tess); and PROMO (http://alggen.lsi.upc.es/cgi-bin/promo_v3/promo/promoinit.cgi?dirDB=TF_8.3). Changes predicted by all 3 packages were then considered.

### HaploReg analysis

The regulatory potential of SNPs in LD to rs4253238 was determined using HaploReg 2 (http://www.broadinstitute.org/mammals/haploreg/haploreg.php). HaploReg makes use of an expanded library of SNPs (based on dbSNP 137), motif instances [based on pulse-width modulations discovered from the Encyclopedia of DNA Elements (ENCODE) experiments], enhancer annotations (adding 90 cell types from the Roadmap Epigenome Mapping Consortium), and eQTLs (from the GTex eQTL browser; ref. 33).

### Haplotype analyses

Linkage disequilibrium analysis was carried out using Haploview 4.2 (HapMap merged versions 1, 2, and 3, release 27 on CEU population; region 4:18737100–18741700; *KLKB1* gene build ENSG00000164344; ref. 34).

### KLKB1 activity assay

Serum samples (5 μl) were added in duplicate to 50 μl of the H-Pro-Phe-Arg-AMC (Bachem, Bubendorf, Liestal, Switzerland) substrate in activity buffer. After a 5-min 25°C incubation, fluorescence was recorded (excitation 380 nm; emission 460 nm) using a Flexstation 3 microplate reader over 5 min. This assay was validated by the addition of 2.5 μg of human plasma extracted KLKB1 (Calbiochem-Merck, Nottingham, UK) to 50 μl of the substrate in activity buffer.

### Cell culture and collection of protein lysate

Normal human bronchial epithelial cells (NHBECs; Lonza, Wokingham, UK) passage 3, from 2 male donors (8), were cultured in fresh growth factor-supplemented medium (BEGM; Lonza), excluding gentamicin and amphotericin-B, in 6-well plates as described previously (8). BEGM was changed to basal medium 24 h prior to stimulation with KLKB1 (Calbiochem-Merck). Cells were incubated for 4 and 24 h. Protein lysates were collected by the addition of 100 μl of X1 SDS loading dye directly to cells, followed by manual scraping and lysate recovery. Experiments were repeated 3 times/donor.

### Quantitative PCR

Levels of total uPAR mRNA in primary HBECs treated with and without KLKB1 (Calbiochem-Merck) were determined using qPCR *via* the TaqMan method as described previously (35). RNA was collected and isolated from cells using the Qiagen RNeasy kit (Qiagen, Manchester, UK) and cDNA synthesized using the SuperScript II Reverse Transcriptase kit using random hexamers (Invitrogen, Paisley, UK).

### Western blotting

Cell lysates were separated by 10% SDS-PAGE under reducing conditions. Proteins were transferred to a PVDF membrane and probed using the anti-uPAR D_I_ antibody IIIF10 (Santa Cruz Biotechnology, Heidelberg, Germany) and the anti-uPAR polyclonal antibody BAF807 (R&D Systems). Detection was *via* enhanced chemiluminescence (Amersham Biosciences, Little Chalfont, UK). Membranes were stripped using Restore Western blot stripping buffer (Thermo Fisher Scientific, Cramlington, UK) and reprobed for loading controls (β-actin; ref. 8). Protein densitometry used ImageJ 1.41 (U.S. National Institutes of Health, Bethesda, MD, USA; http://rsbweb.nih.gov/ij/). For statistical analysis, data were normalized to β-actin and exposure levels.

### KLKB1-mediated scuPAR digest

We next treated 200 ng of recombinant human uPAR (R&D Systems) with 250 ng of KLKB1 at 37°C for 24 h. A protease control (protease inhibitor cocktail mix; Roche, Welwyn Garden City, UK), and a cleavage control (substitution of KLKB1 with 108 ng of the known uPAR protease chymotrypsin; Roche) were used.

### Preparation and transfection of plasmids

The pcDNA3+muPAR plasmid has been described previously (8). The open reading frame of membrane uPAR (NM_002659.3) minus the sequence coding for the GPI anchor was amplified using primers including a consensus Kozak sequence and restriction enzyme sites (5′ primer: 5′-ACTTGAATTCGCCACCATGGGTCACCCGCCGCT-3′; 3′ primer: 5′-ACTTCTCGAGTTAACAGCCACTTTTAGTACAGC-3′). The PCR product was cloned into pCR4-TOPO (Invitrogen) and then subcloned into pcDNA3 (Promega, Southampton, UK) using appropriate restriction enzymes and T4 ligase (Promega). The scuPAR plasmid was sequence verified before transfection.

### Wound-healing assay

NHBECs were cultured as above in 6-well plates. Cells were transfected with muPAR- or scuPAR-overexpressing plasmids (8) at 50% confluence using Fugene6 at a ratio of Fugene to DNA of 3:2. Wound healing was carried out as described previously (8). Percentage area healed was calculated using CellProfiler (36).

### Proliferation assay

NHBECs were cultured in 96-well plates and transfected as above. At 20 h post-transfection, cells were exposed to a 0.5 mg/ml dimethyl-thiazolyl-diphenyl-tetrazolium solution (Sigma-Aldrich, Dorset, UK) at 37°C for 4 h. Crystals were dissolved in isopropanol and absorbance measured at 570 nm (background subtraction at 690 nm) using a Flexstation 3 microplate reader.

### Statistical analyses

Statistical analyses were carried out using SPSS (PASW) 16.0 (SPSS Inc., Chicago, IL, USA) or GraphPad Prism 5.03 (GraphPad, San Diego, CA, USA). Potential continual and binary variable confounders for serum scuPAR level analyses were systematically investigated in the regression model. Variables included in the final model had a statistically significant effect on scuPAR levels, *i.e*., age (in years at time of sampling), height (in meters at time of sampling), weight (in kilograms at time of sampling), and smoking (pack-year value). Gender and geographical locations were not identified as confounders. Investigations into the association of serum scuPAR levels with disease phenotypes were carried out using Mann-Whitney and Kruskal-Wallis tests, followed by secondary confirmatory analyses using linear regression, with the inclusion of identified confounders. Duplication of GWAS analysis in the COPD cohort was carried out in PLINK (additive model) on the log_10_ transformed uPAR values. Data from grouped analyses were analyzed using either a paired 1-way ANOVA or a 2-tailed paired *t* test carried out in PRISM.

## RESULTS

### Serum scuPAR levels are elevated in asthma and COPD

**Figure 1.**
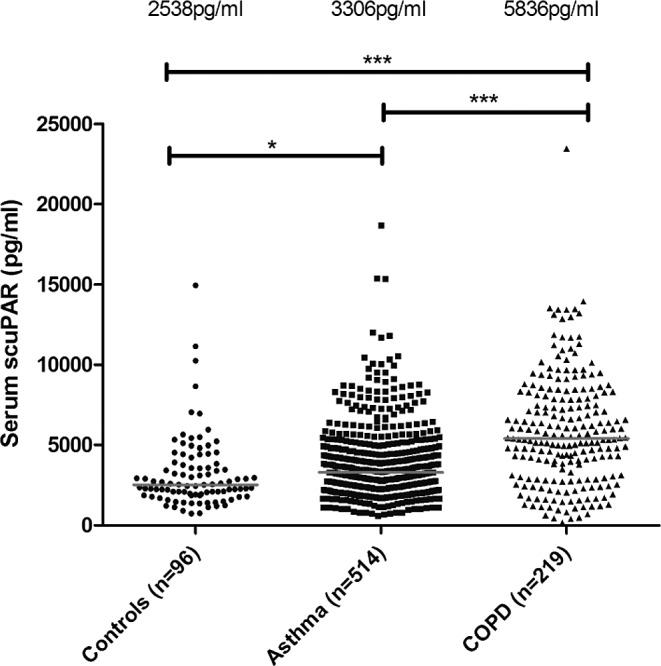
Elevated scuPAR in serum from patients with asthma or COPD. Median levels are elevated in asthma (3306 pg/ml) and COPD (5836 pg/ml) when compared to controls (2538 pg/ml). Patients with COPD also had a higher level of scuPAR than patients with asthma. Lines denote median scuPAR levels. *P = 1 × 10^−3^, ***P < 1 × 10^−5^.

Median serum scuPAR levels in 514 patients with asthma (3306 pg/ml) and 219 patients with COPD (5844 pg/ml) were elevated *vs.* 96 control subjects (2538 pg/ml) (*P*=0.001 and *P*=1×10^−4^, respectively; **Fig. 1**). Subjects were selected based on the presence of full clinical characteristics (see **Table 1**). There was a significantly elevated level of scuPAR in COPD *vs.* those subjects with asthma (*P*<0.0001; Fig. 1). All analyses were corrected for the identified confounders of age, height, weight, and smoking (pack-years).

**Table 1.** Population demographics for patient serum samples

Parameter	Control	Asthma	COPD
Maximum number	104	514	219
Percentage male	37.5%	44.9%	62.7%
Mean age (yr)*	50 ± 8	40 ± 12	68 ± 8
Mean height (m)	1.71 ± 0.08	1.74 ± 0.09	1.67 ± 0.88
Mean weight (kg)	76.35 ± 12.1	78.12 ± 15.7	70.23 ± 18.1
Mean smoking (pack-yr)*	10.50 ± 11.69	4.95 ± 9.58	46.87 ± 26.72
FEV_1_ pre- (%Pred)*	100.94 ± 10.99	80.71 ± 20.70	41.87 ± 18.52
FEV_1_ post- (%Pred)*	104.78 ± 11.32	92.42 ± 18.73	47.53 ± 19.39
FEV_1_/VC pre-*	0.80 ± 0.05	0.70 ± 0.14	0.45 ± 0.13
FEV_1_/VC post-*	0.82 ± 0.04	0.76 ± 0.12	0.45 ± 0.12
Reversibility (% change in FEV_1_ from baseline to salbutamol)*	3.91 ± 4.43	11.72 ± 7.10	6.64 ± 3.29
BHR (PC_20_; mg/ml)	>32	<32	n/a

Subjects were selected from the total pool based on presence of complete clinical data for phenotype analyses or genotype-specific quality control that excluded subjects. Pre- indicates measurements taken prior to the administration of a bronchodilator (salbutamol). Post- indicates measurements taken after the administration of a bronchodilator (salbutamol). BHR, bronchial hyperresponsiveness. Values are means ± sd.

* *P* < 0.01 across groups.

### Genome-wide association identifies rs4253238 as a determinant of scuPAR serum levels

**Figure 2.**
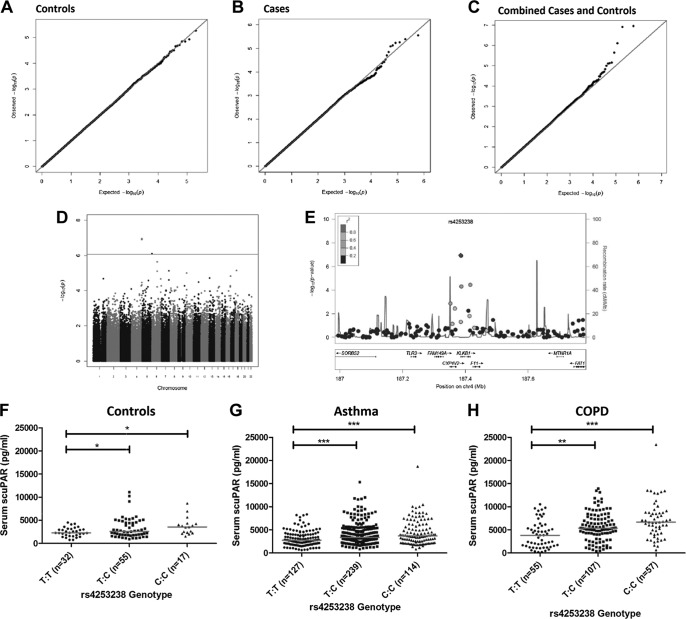
Serum scuPAR is associated with the *KLKB1* SNP rs4253238. *A*, *B*) QQ plot for genome-wide analyses in control (*n*=104; *A*) and asthma (*n*=480; *B*) populations. The QQ plots identify a modest deviation in the disease (asthma) population. *C*) In an analysis of the asthma and control populations (*n*=584), the QQ plot reveals that measured SNP log *P* values deviate significantly from the expected trend denoted by the red trend line. *D*) Manhattan plot identifies a single region as the genome-wide significant in a combined asthma/control population analysis (*P*=1.69×10^−7^). This region contains 2 SNPs (rs425328 and rs1912826) that lie in the intergenic space between the genes *KLKB1*, *FXI*, and *CYP4V2. E*) Region plot investigation identifies SNP rs4253238 as the main associated SNP in this region, with supporting evidence. *F–H*) Analysis of the relationship bewween rs4253238 and serum scuPAR in the COPD cohort (*n*=219; *H*) reveals that the same direction of effect exists across the control (*F*) and asthma (*G*) populations. These differences were statistically significant (*P*<0.001). Red lines denote median scuPAR levels. *P < 0.05, **P < 0.001, ***P < 0.0001.

We investigated 295,196 SNPs for association with scuPAR levels in serum. Quantile-quantile plots for controls (*n*=104) and patients with asthma (*n*=480) identified a lack of deviation of observed *P* values from the expected *P* values (**Fig. 2*A*, *B***; inflation factor λ=0.996 and 1.003, respectively). In individual data sets, no SNP achieved genome-wide significance, as defined by the Bonferroni method (*P*<*1*.69×10^−7^). Analysis carried out on the control and asthma result data sets (*n*=584, λ=1.009) identified a region at 4q35 containing genome-wide significant associations for SNPs rs4253238 and rs1912826 [Fig. 2*C, D*; *P*(fixed effect)=1.13×10^−7^]. These SNPs are in near complete linkage disequilibrium (LD; *D*′=0.99; *R*^2^=0.94) in the GWAS population and so were considered as a single region of variation, represented by rs4253238 for the remainder of this study. The 4q35 region corresponds to the promoter/5′ coding region of the gene for human plasma kallikrein (*KLKB1*; previously known as *KLK3*).

### rs4253238 is associated with scuPAR levels in a COPD population

SNP rs4253238 was genotyped in a COPD cohort (*n*=219) and was associated with serum scuPAR levels (*P*=5.34×10^−7^; *B*=0.16812 for log_10_-transformed uPAR levels and additive allele coding). The association identified the same direction of effect on scuPAR levels as in the asthma/control meta-analysis [median level for genotypes C:C (6654 pg/ml) > T:C (5435 pg/ml) > T:T (4412 pg/ml) (Fig. 2*F–H*), minor allele: C; Caucasian (HapMap CEU) minor allele frequency (MAF)=0.496 (Fig. 2*H*)].

A meta-analysis including the control, asthma, and COPD populations (*n*=803) further confirmed association between rs4253238 and serum scuPAR (*P*=5.037×10^−12^; *B*=0.0879). A significant degree of heterogeneity of the effect between the studies (*P*=0.02) was observed and reflected a more pronounced effect by rs4253238 on serum scuPAR in COPD (*B*=0.16812) than in patients with asthma (*B*=0.06972) or controls (*B*=0.09129).

### rs4253238 is in linkage disequilibrium with multiple potentially functional KLKB1 SNPs

**Figure 3.**
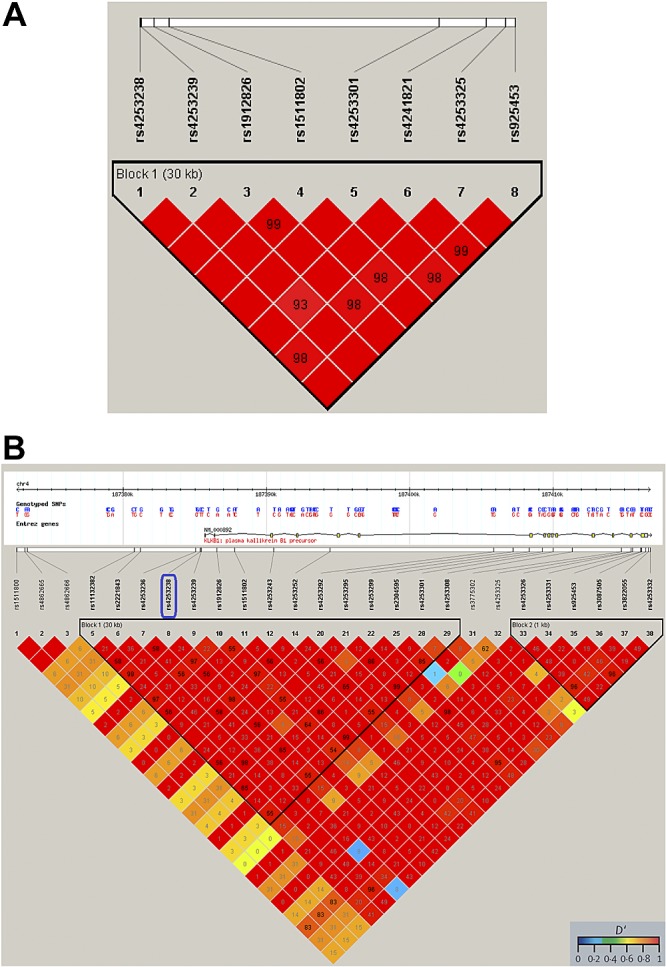
SNP rs4253238 is in LD with other *KLKB1* SNPs. A) LD plot of the GWAS identified SNP rs4253238 identifies a block of LD between this SNP and other SNPs present in the *KLKB1* gene, in the control/asthma data set (*n*=584). *B*) LD plot generated with Haploview, utilizing HapMap data selected for the CEU (Utah residents with northern and western European ancestry) population, confirms the presence of a block of LD for SNP rs4253238 with SNPs in the 5′ region of the *KLKB1* gene. Numbers identify the *R*^2^ values up to a maximum of 100. The color scheme denotes the level of LD as defined by *D*′. Similar plots identify no LD with regions downstream of rs4253238 (data not shown).

SNP rs4253238 is in LD with SNPs present in the *KLKB1* 5′UTR and coding regions in the HapMap Project (34) Caucasian populations (Utah residents with ancestry from northern and western Europe (*n*=180), but not with SNPs in other genes (**Fig. 3*B***). The HaploReg online tool (33) identified 31 SNPs in LD with rs4253238 that have potential regulatory function on *KLKB1* gene expression using the 1000 genomes' phase 1 data (see Supplemental Fig. S1).

### A near genome-wide significant hit exists on the *FXII* gene but is not observed in a COPD cohort

A second SNP located on chromosome 5 (rs2731672; MAF=0.190; HapMap CEU population), located in the 3′ region of the factor XII gene, just failed to achieve genome-wide significance (*P*=7.83×10^−7^; *B*=0.0782 for log_10_ transformed uPAR level and additive allele coding). However, no association was found between the SNP and serum scuPAR in the COPD cohort (*P*>0.05). This may be explained by the fact that the additive effect of this SNP is 2 times larger in the controls (*B*=0.1414) than in the patients with asthma (*B*=0.06204), identifying a possible effect unique to nondiseased individuals. However, as we did not observe this association in an independent cohort (COPD: *B*=0.00016 and *P*=0.997), we therefore focused on the locus defined by SNP rs4253238.

### An *in silico* eQTL analysis does not identify SNP rs4253238 as being associated with uPAR mRNA

Comparisons between data generated from the protein GWAS and *in silico* data from the mRNA by SNP browser 1.0.1 (32) identified that rs4253238 did not alter uPAR mRNA levels in this lymphoblastoid database. The polymorphism located at rs4253238 was also not found to regulate uPAR levels in a mRNA-based investigation to determine genetic variants that affect gene expression in human lung tissue (37). These data suggested that the genetic association for rs4253238 did not involve changes in mRNA and may be a post-translational mechanism.

### rs4253238 does not alter uPAR mRNA in a primary epithelial cell population

**Figure 4.**
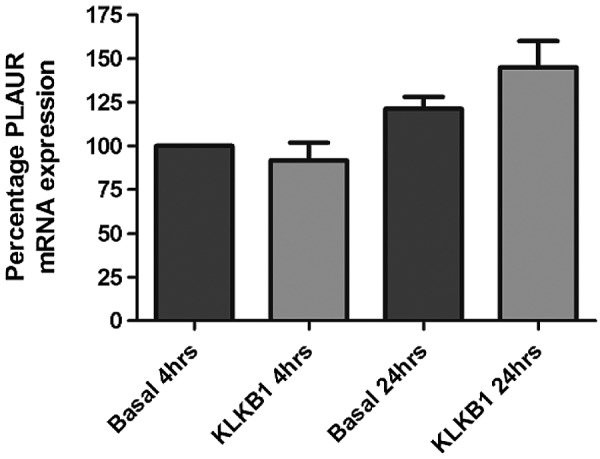
KLKB1 does not alter *PLAUR* mRNA expression. Addition of KLKB1 to a population of primary HBECs did not alter the rate of total *PLAUR* mRNA expression either at 4 or 24 h postexposure. This suggests that changes in circulating levels of scuPAR driven by KLKB1 are not driven by changes in mRNA expression patterns.

Expanding on the *in silico* analysis, comparisons of membrane uPAR mRNA levels in primary human bronchial epithelial cells treated with and without KLKB1 confirmed that KLKB1 did not modulate membrane uPAR expression at the mRNA level (**Fig. 4**).

### rs4253238 genotype is associated with human plasma kallikrein activity in serum

**Figure 5.**
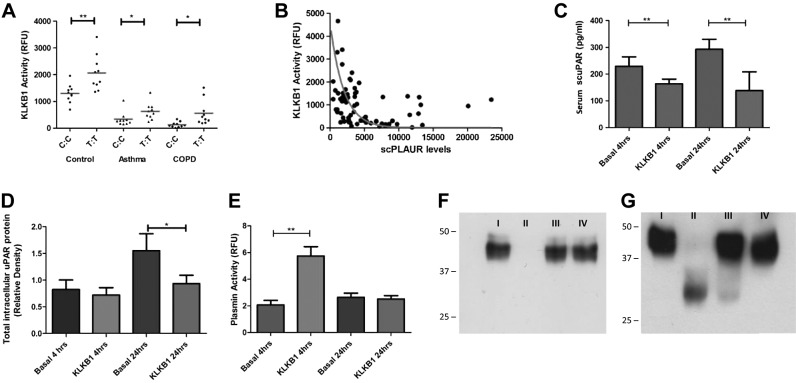
SNP rs4253238 modulates KLKB1 enzymatic activity, which, in turn, determines scuPAR levels. *A*) KLKB1 activity assay identified an elevation of KLKB1 activity with the rs4253238 T:T genotype in control (*P*=0.004), asthma (*P*=0.034), and COPD (*P*=0.011) populations. KLKB1 activity was also found to be reduced in the 2 diseased populations when compared to controls (*P*=1×10^−4^). *B*) KLKB1 activity was found to be inversely correlated with scuPAR levels regardless of whether the subjects were controls, patients with asthma, or patients with COPD (*P*=1×10^−4^; *R*^2^=0.278). *C*, *D*) Exposure of NHBECs to plasma-extracted human KLKB1 results in reduced levels of scuPAR in cell supernatants at 4 and 24 h (*P*=0.015 and *P*=0.029, respectively; *C*), while total intracellular protein was decreased at 24 h (*P*=0.05; *D*). *E*) Within the same cell system, plasmin was activated by KLKB1 at 4 h, but not at 24 h (*P*=5×10^−3^), identifying the need for a cell-free system to investigate the direct effect of *KLKB1* on scuPAR. *F*, *G*) Staining of recombinant human uPAR (ruPAR) digested with KLKB1 with the uPAR D_I_-specific monoclonal antibody IIIF10 (*F*) and the polyclonal antibody BAF 807 (*G*) reveals that KLKB1 proteolytically cleaves the ruPAR molecule at the D_I_ region (*F*), leaving behind a fragment of ∼30 kDa, comparable to that formed following digestion of a known uPAR proteolytic enzyme, chymotrypsin (*P*<0.001). I, ruPAR; II, ruPAR + KLKB1; III, ruPAR + chymotrypsin; IV, ruPAR + KLKB1 + protease inhibitor cocktail. *P < 0.05, **P < 0.001.

Using a validated activity assay, the *KLKB1* rs4253238 genotype was associated with KLKB1 activity using a fluorescent substrate in 10 randomly chosen homozygote subjects (TT *vs.* CC) from each of the control, asthma, and COPD populations (**Fig. 5*A***). Presence of the major (T) allele was associated with increased KLKB1 activity in the control (*P*<0.01), asthma (*P*<0.01), and COPD populations (*P*<0.001). Notably, KLKB1 activity was decreased in diseased patients when compared to controls (*P*<0.001). Serum KLKB1 activity was inversely correlated to serum scuPAR levels (*P*<1×10^−4^; *R*^2^=0.278; Fig. 5*B*).

### KLKB1 exposure reduces scuPAR protein levels in bronchial epithelial cells and increases plasmin activity

We next determined the biological activity of KLKB1 on primary HBECs, which are key effector cells for remodeling in asthma and COPD (38). HBECs from 2 Caucasian donors were cultured in the presence and absence of KLKB1; scuPAR levels in supernatants of HBECs treated with KLKB1 were decreased at 4 and 24 h (*P*=0.015; *P*=0.029; Fig. 5*C*). HBEC total intracellular uPAR protein levels (muPAR and suPAR) were decreased following KLKB1 exposure at 24 h (*P*<0.05; Fig. 5*D*).

To investigate whether KLKB1 affects scuPAR through modulation of the plasminogen activation system we carried out a plasmin activity assay, identifying a ∼3-fold increase in HBEC supernatant plasmin activity at 4 h (*P*=0.005), but not at 24 h after KLKB1 addition (Fig. 5*E*).

### KLKB1 cleaves recombinant scuPAR in a cell-free system

KLKB1 induces loss of the uPAR D_I_ epitope, as evidenced by Western blotting using a D_I_-specific uPAR monoclonal antibody (IIIF10) on recombinant uPAR (ruPAR) incubated with KLKB1 (Fig. 5*F*). No other fragments were observed. Western blot analysis using the uPAR polyclonal antibody BAF807 identified the KLKB1-dependant loss of full-length ruPAR and production of a ∼30-kDa fragment (Fig. 5*G*). N-terminal sequencing identified the location of this fragment's N-terminal as adjacent to uPAR D_III_ (sequence THEP). KLKB1-mediated uPAR cleavage was blocked through the use of a protease inhibitor (Fig. 5*F*, *G*). Incubation of recombinant uPAR with KLKB1 also rendered uPAR undetectable by ELISA (see Supplemental Fig. S2).

### KLKB1 reverses scuPAR-mediated effects on bronchial epithelial cell proliferation and wound repair

**Figure 6.**
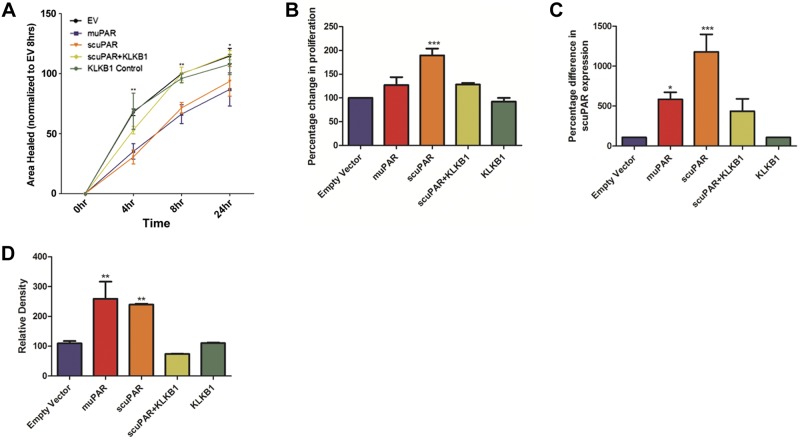
scuPAR-driven alterations in HBEC functions are attenuated by KLKB1. *A*) scuPAR attenuates wound repair in a scratch-wound model when compared to the empty vector control at the 4 h (*P*<0.001) and 8 h (*P*<0.05) time points, an effect mirrored by muPAR (*P*<0.01). *B*) scuPAR also increases bronchial epithelial cell proliferation (*P*=2×10^−4^), while muPAR does not alter the rate of proliferation. *C*, *D*) Addition of KLKB1 negates all scuPAR-driven effects. Overexpression of scuPAR and muPAR by the transiently transfected primary HBECs was confirmed both in cell supernatant by ELISA (*P*=2.6×10^−3^; *C*) and in cell lysate by Western blotting (*P*=1.3×10^−3^; *D*), where KLKB1 was found to reduce scuPAR overexpression (*P*<0.001). *P < 0.05, **P < 0.001, ***P < 0.0001.

The effect of scuPAR on epithelial cell migration and proliferation in relation to muPAR-driven effects was investigated in HBECs recombinantly expressing an engineered form of scuPAR (lacking GPI) or full-length muPAR. Overexpression of scuPAR or muPAR resulted in excess scuPAR in the cell supernatant and attenuation of HBEC wound repair in a scratch-wound model (*P*<0.001; **Fig. 6*A***) while scuPAR alone increased cell proliferation (*P*=2×10^−4^; Fig. 6*B*). All scuPAR-mediated effects were negated in the presence of KLKB1 (Fig. 6*A, B*).

## DISCUSSION

In the current study we aimed to investigate the expression profile of scuPAR in asthma and COPD and use genetics to identify novel regulatory mechanisms. In the initial part of this study we provide the first evidence that serum-circulating scuPAR is elevated in asthma and COPD. This identifies these populations as suitable cohorts for genome-wide analyses carried out to identify novel regulatory mechanisms in disease. Using a genome-wide protein QTL mapping approach we then identify a novel regulatory mechanism of scuPAR expression, where scuPAR serum levels are inversely correlated with serum KLKB1 activity, which is driven by SNP rs4253238. Finally we identified that KLKB1 modulation of scuPAR has implications for scuPAR-driven effects on cell function, including both proliferation and migration.

Our data suggest that serum scuPAR is associated with asthma and COPD, with higher overall levels of scuPAR detected in COPD, a highly neutrophilic disease characterized by less airway hyperresponsiveness and reversibility when compared to asthma (39). This extends our previous work, where we identified an elevated level of uPAR in the airway epithelium in asthma (8), and suggests a systematic increase in this integral protein.

scuPAR is thought to be generated from membrane uPAR by the activity of a number of different enzymes, including phospholipases C and D, which cleave the receptor's GPI anchor (5, 40). Similarly, a number of different proteases, including pepsin, MMP-12, chymotrypsin, human airway trypsin-like protease, cathepsin G, and urokinase, can cleave the receptor linker region between D_I_ and D_II_, (residues 83 to 89) to generate D_I_- and D_II/III_-containing proteins (41–45). However, at this time, the relevance of these or any other cleavage mechanism ultimately determining serum levels of scuPAR (and other enzymatically generated forms) remains to be clarified. To define novel regulatory mechanisms for scuPAR we utilized a protein QTL-mapping approach. This decision was carried out based on previous knowledge that protein QTL mapping can be successfully employed to identify interactive protein networks in humans and discover novel biological mechanisms (46).

We identified rs4253238 as a promoter polymorphism with *KLKB1*, a chromosome 4q34-35 gene encoding prekallikrein (47). Prekallikrein, cleaved by activated factor XII, with high-molecular-weight kininogen as a cofactor, forms human plasma kallikrein (KLKB1; ref. 47). Interestingly, an SNP (rs2731672) in proximity (5897 bp) to the factor XII gene, nearly achieved genome-wide significance in this study, suggesting multiple functional SNPs in this pathway. KLKB1 is a serine protease that circulates at a serum concentration of 50 μg/ml (48) and participates in the contact activation system of coagulation (49, 50) and plasmin activation (51). Presence of the C allele of rs4253238 results in the loss of 3 putative transcription factor binding domains: neurogenic differentiation 1 (NeuroD1), Kruppel-like C2H2 zinc finger, and PAR/pZIP family, suggesting a role for this SNP in *KLKB1* regulation. High LD existed between rs4253238 and other *KLKB1* SNPs, but not any of the genes adjacent to *KLKB1*. However, haplotype analysis identified that rs4253238 may be simply a marker for an alternate regulatory SNP. This hypothesis was further validated by an *in silico* analysis of the potential functional effects, where a number of SNPs in LD were shown to have gene expression regulatory potential. A link between rs4253238 and KLKB1 activity was confirmed through a serum KLKB1 protease activity assay, with the C allele associated with lower KLKB1 activity, forming an inverse KLKB1 activity/uPAR relationship. We confirmed this relationship in the supernatant and total intracellular protein of primary bronchial epithelial cells (a major source of uPAR in the airways). Although the relationship between KLKB1 and uPAR levels was not unique to diseased populations, we identified overall reduced KLKB1 activity in the asthma and COPD populations, which may at least in part contribute to the elevated scuPAR levels detected in these disease populations. Therefore, although KLKB1 regulation of scuPAR is a regulatory pathway observed in both disease and control populations, results from the activity assay and GWAS heterogeneity tests allow us to propose a significantly reduced KLKB1 activity in obstructive lung disease. This would significantly contribute to the elevated levels of scuPAR in serum detected in this study and in multiple other diseases in previous investigations (22).

To further define the mechanism driving KLKB1 regulation of scuPAR levels and to remove any possible confounding effect by the uPAR GPI anchor protease plasmin, which has been shown to be elevated by KLKB1 (51), we carried out experiments using a cell-free system. Incubation of recombinant uPAR with KLKB1 resulted in loss of D_I_, while N-terminal sequencing identified a cleavage site adjacent to D_III_. Regulation of scuPAR by KLKB1 *via* a direct proteolytic mechanism correlates well with a previous investigation into relationships between human tissue kallikrein (hK4), a nonhomologous relative of KLKB1, and uPAR (52). Recently reported proteolytic effects of KLKB1 on other molecules such as endothelin-1 and adrenomedullin (53) also lend weight to this argument. We therefore conclude that KLKB1 regulates uPAR levels by acting as an uPAR proteolytic enzyme, in a plasmin-independent manner.

Disease and biological roles for uPAR have already been described at the cellular level. Increased uPAR expression has been determined at the leading edge of reepithelializing wounds (8), while uPAR-dependent activation of TGFβ and hepatocyte growth factor/scatter factor (HGF/SF) is known to increase the rate of proliferation in epithelial cells (54). We therefore set out to determine whether the soluble cleaved form of the receptor has its own independent role on epithelial function and whether KLKB1 is capable of inhibiting these effects.

Soluble cleaved uPAR has been shown to be biologically active in epithelial cells (8). In this study we have extended this observation, identifying a physiological role for scuPAR in HBECs, where scuPAR modulates proliferation and wound repair. Soluble cleaved uPAR attenuates the rate of wound repair to a similar degree as muPAR (8) and independently increases the rate of proliferation, with addition of KLKB1 successfully inhibiting these effects. This is in good agreement with previously published work on the effect of KLKB1, on epithelial wound repair (55). Here Lund *et al.* (55) describe how inhibition of KLKB1 activity leads to strong attenuation of wound repair in a mouse model independently of both uPA and tPA. Although unreported by the authors, it is likely that the reported effect may be due to attenuated cleavage of uPAR. As both attenuated wound repair and increased proliferation are important contributing factors toward airway remodeling (56, 57), it is likely that modulation of these cell mechanisms by scuPAR and KLKB1 may contribute to disease where epithelial function plays a crucial role, such as obstructive lung disease, FSGS, and certain cancers.

It is likely that the rs4253238 SNP may also be of relevance to other KLKB1-driven disease processes through its modulation of KLKB1 activity. KLKB1 has a known proteolytic function, activating a number of proteins including bradykinin and blood clotting factor XII (58). Interestingly, a *FXII* SNP demonstrated near genome-wide significance with serum scuPAR levels in the current study and FXII has been shown to interact with uPAR to stimulate ERK1/2 and Akt signaling pathways (59). KLKB1 has also been implicated in the stimulation of neutrophil chemotaxis, aggregation, and oxygen consumption (60). The majority of known KLKB1 activity is attributed to the cleavage of these 2 substrates/cofactors and regulation of neutrophil function, including effects on inflammation, vascular function, blood pressure regulation, and nociceptive responses (61). This could be of relevance to multiple diseases including lung disease, where both neutrophilic inflammation, *e.g.*, severe asthma and COPD, and the fibrinolytic/coagulation cascade, *e.g.*, asthma, COPD and idiopathic pulmonary fibrosis, have been implicated.

**Figure 7.**
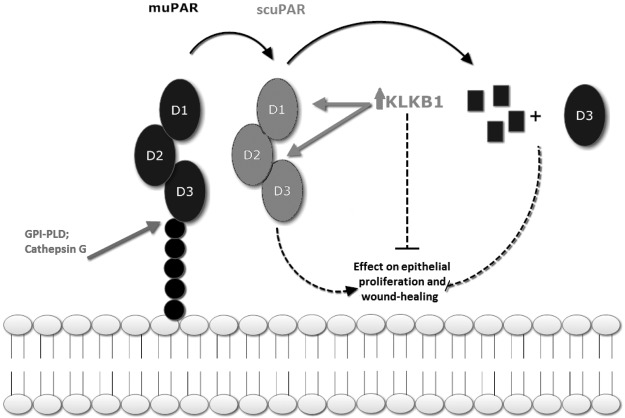
Role of KLKB1 in regulating scuPAR. Membrane-bound uPAR (muPAR) is a 3-globular-domain (D_I_, D_II_, and D_III_) protein attached to the cell membrane *via* a GPI anchor. D_I_ and D_II_ bind to the ligand urokinase, while D_III_ is known to interact with cell-bound integrins. The GPI anchor is susceptible to both glycolytic and lipolytic cleavage, resulting in the release of a cleaved full-length soluble receptor, soluble cleaved uPAR, in which all domains are intact. Proteolytic action carried out by KLKB1, which is elevated in carriers of the rs4253238 T allele, disrupts the receptor's globular domains, inhibiting receptor-driven effects on bronchial epithelial cell proliferation and wound healing. GPI-PLD, GPI-specific phospholipase D.

In summary, we identified elevated circulating serum scuPAR in asthma and COPD. We completed a human genome-wide protein QTL map, identifying a post-translational serum scuPAR regulatory mechanism through an association with *KLKB1* promoter SNP rs4253238. The rs4253238-associated protein KLKB1 regulates scuPAR levels through KLKB1-mediated proteolytic cleavage at the protein's D_I_ region. This proteolytic mechanism in turn modulates alteration in epithelial wound repair and proliferation (Fig. 6). Cleavage also results in scuPAR being unable to interact with integrins (62, 63) or sequester circulating urokinase (2), effecting the modulation of uPAR associated diseases (**Fig. 7**). Although common to both the diseased and nondiseased populations, we identify differences in activity between diseased (COPD/asthma) and control populations. SNP rs4253238 is also a prevalent SNP (MAF=0.496; HapMap CEU population) making it a potential widespread regulator of scuPAR with relevance in multiple human conditions, although further studies are needed to identify the SNPs in the rs4253238 LD block that are functionally relevant. We therefore hypothesize that KLKB1-mediated reduction of scuPAR as identified in this study may play an important role in scuPAR-associated diseases, such as prostate (10) and breast (9) cancers, epilepsy (11), cardiovascular disease (12), and FSGS (24).

## Supplementary Material

Supplemental Data

## References

[1] Blasi F. Sidenius N. (2010) The urokinase receptor: focused cell surface proteolysis, cell adhesion and signaling. FEBS Lett. 584, 1923–1930 20036661 10.1016/j.febslet.2009.12.039

[2] Blasi F. Carmeliet P. (2002) uPAR: a versatile signalling orchestrator. Nat. Rev. Mol. Cell Biol. 3, 932–943 12461559 10.1038/nrm977

[3] Ploug M. Behrendt N. Lober D. Dano K. (1991) Protein structure and membrane anchorage of the cellular receptor for urokinase-type plasminogen activator. Semin. Thromb. Hemost. 17, 183–193 1665583 10.1055/s-2007-1002608

[4] Plesner T. Ploug M. Ellis V. Ronne E. Hoyer-Hansen G. Wittrup M. Pedersen T. L. Tscherning T. Dano K. Hansen N. E. (1994) The receptor for urokinase-type plasminogen activator and urokinase is translocated from two distinct intracellular compartments to the plasma membrane on stimulation of human neutrophils. Blood 83, 808–815 8298141

[5] Ploug M. Ronne E. Behrendt N. Jensen A. L. Blasi F. Dano K. (1991) Cellular receptor for urokinase plasminogen activator. Carboxyl-terminal processing and membrane anchoring by glycosyl-phosphatidylinositol. J. Biol. Chem. 266, 1926–1933 1846368

[6] Stewart C. E. Sayers I. (2009) Characterisation of urokinase plasminogen activator receptor variants in human airway and peripheral cells. BMC Mol. Biol. 10, 75 19638192 10.1186/1471-2199-10-75 PMC2724484

[7] Barton S. J. Koppelman G. H. Vonk J. M. Browning C. A. Nolte I. M. Stewart C. E. Bainbridge S. Mutch S. Rose-Zerilli M. J. Postma D. S. Maniatis N. Henry A. P. Hall I. P. Holgate S. T. Tighe P. Holloway J. W. Sayers I. (2009) PLAUR polymorphisms are associated with asthma, PLAUR levels, and lung function decline. J. Allergy Clin. Immunol. 123, 1391–1400, e1317 19443020 10.1016/j.jaci.2009.03.014

[8] Stewart C. E. Nijmeh H. S. Brightling C. E. Sayers I. (2011) uPAR regulates bronchial epithelial repair in vitro and is elevated in asthmatic epithelium. Thorax 67, 477–487 22139533 10.1136/thoraxjnl-2011-200508 PMC3358731

[9] Raghu H. Sodadasu P. K. Malla R. R. Gondi C. S. Estes N. Rao J. S. (2010) Localization of uPAR and MMP-9 in lipid rafts is critical for migration, invasion and angiogenesis in human breast cancer cells. BMC Cancer 10, 647 21106094 10.1186/1471-2407-10-647 PMC3002355

[10] Gutova M. Najbauer J. Frank R. T. Kendall S. E. Gevorgyan A. Metz M. Z. Guevorkian M. Edmiston M. Zhao D. Glackin C. A. Kim S. U. Aboody K. S. (2008) Urokinase plasminogen activator and urokinase plasminogen activator receptor mediate human stem cell tropism to malignant solid tumors. Stem Cells 26, 1406–1413 18403751 10.1634/stemcells.2008-0141

[11] Bruneau N. Szepetowski P. (2011) The role of the urokinase receptor in epilepsy, in disorders of language, cognition, communication and behavior, and in the central nervous system. Curr. Pharm. Des. 17, 1914–1923 21711233 10.2174/138161211796718198

[12] Xu J. Li W. Bao X. Ding H. Chen J. Zhang W. Sun K. Wang J. Wang X. Wang H. Yu H. Song W. Ma W. Zhang L. Wang C. Wang D. Hui R. (2010) Association of putative functional variants in the PLAU gene and the PLAUR gene with myocardial infarction. Clin. Sci. (Lond.) 119, 353–359 20518747 10.1042/CS20100151

[13] Ploug M. (2003) Structure-function relationships in the interaction between the urokinase-type plasminogen activator and its receptor. Curr. Pharm. Des. 9, 1499–1528 12871065 10.2174/1381612033454630

[14] Tan X. Egami H. Nozawa F. Abe M. Baba H. (2006) Analysis of the invasion-metastasis mechanism in pancreatic cancer: involvement of plasmin(ogen) cascade proteins in the invasion of pancreatic cancer cells. Int. J. Oncol. 28, 369–374 16391791

[15] Aguirre-Ghiso J. A. Liu D. Mignatti A. Kovalski K. Ossowski L. (2001) Urokinase receptor and fibronectin regulate the ERK(MAPK) to p38(MAPK) activity ratios that determine carcinoma cell proliferation or dormancy in vivo. Mol. Biol. Cell 12, 863–879 11294892 10.1091/mbc.12.4.863 PMC32272

[16] Thuno M. Macho B. Eugen-Olsen J. (2009) suPAR: the molecular crystal ball. Dis. Markers 27, 157–172 19893210 10.3233/DMA-2009-0657 PMC3835059

[17] Sidenius N. Sier C. F. Ullum H. Pedersen B. K. Lepri A. C. Blasi F. Eugen-Olsen J. (2000) Serum level of soluble urokinase-type plasminogen activator receptor is a strong and independent predictor of survival in human immunodeficiency virus infection. Blood 96, 4091–4095 11110678

[18] Xiao W. Hsu Y. P. Ishizaka A. Kirikae T. Moss R. B. (2005) Sputum cathelicidin, urokinase plasminogen activation system components, and cytokines discriminate cystic fibrosis, COPD, and asthma inflammation. Chest 128, 2316–2326 16236890 10.1378/chest.128.4.2316

[19] Blasi F. (2011) The urokinase receptor in hematopoietic stem cells mobilization. Curr. Pharm. Des. 17, 1911–1913 21711240 10.2174/138161211796718206

[20] Savva A. Raftogiannis M. Baziaka F. Routsi C. Antonopoulou A. Koutoukas P. Tsaganos T. Kotanidou A. Apostolidou E. Giamarellos-Bourboulis E. J. Dimopoulos G. (2011) Soluble urokinase plasminogen activator receptor (suPAR) for assessment of disease severity in ventilator-associated pneumonia and sepsis. J. Infect. 63, 344–350 21839112 10.1016/j.jinf.2011.07.016

[21] Andersen O. Eugen-Olsen J. Kofoed K. Iversen J. Haugaard S. B. (2008) Soluble urokinase plasminogen activator receptor is a marker of dysmetabolism in HIV-infected patients receiving highly active antiretroviral therapy. J. Med. Virol. 80, 209–216 18098145 10.1002/jmv.21114

[22] Eugen-Olsen J. Andersen O. Linneberg A. Ladelund S. Hansen T. W. Langkilde A. Petersen J. Pielak T. Moller L. N. Jeppesen J. Lyngbaek S. Fenger M. Olsen M. H. Hildebrandt P. R. Borch-Johnsen K. Jorgensen T. Haugaard S. B. (2010) Circulating soluble urokinase plasminogen activator receptor predicts cancer, cardiovascular disease, diabetes and mortality in the general population. J. Intern. Med. 268, 296–308 20561148 10.1111/j.1365-2796.2010.02252.x

[23] Haupt T. H. Petersen J. Ellekilde G. Klausen H. H. Thorball C. W. Eugen-Olsen J. Andersen O. (2012) Plasma suPAR levels are associated with mortality, admission time, and Charlson Comorbidity Index in the acutely admitted medical patient: a prospective observational study. Crit. Care 16, R130 22824423 10.1186/cc11434 PMC3580714

[24] Wei C. El Hindi S. Li J. Fornoni A. Goes N. Sageshima J. Maiguel D. Karumanchi S. A. Yap H. K. Saleem M. Zhang Q. Nikolic B. Chaudhuri A. Daftarian P. Salido E. Torres A. Salifu M. Sarwal M. M. Schaefer F. Morath C. Schwenger V. Zeier M. Gupta V. Roth D. Rastaldi M. P. Burke G. Ruiz P. Reiser J. (2011) Circulating urokinase receptor as a cause of focal segmental glomerulosclerosis. Nat. Med. 17, 952–960 21804539 10.1038/nm.2411 PMC4089394

[25] Stewart C. E. Hall I. P. Parker S. G. Moffat M. F. Wardlaw A. J. Connolly M. J. Ruse C. Sayers I. (2009) PLAUR polymorphisms and lung function in UK smokers. BMC Med. Genet. 10, 112 19878584 10.1186/1471-2350-10-112 PMC2784766

[26] Koppelman G. H. Meyers D. A. Howard T. D. Zheng S. L. Hawkins G. A. Ampleford E. J. Xu J. Koning H. Bruinenberg M. Nolte I. M. van Diemen C. C. Boezen H. M. Timens W. Whittaker P. A. Stine O. C. Barton S. J. Holloway J. W. Holgate S. T. Graves P. E. Martinez F. D. van Oosterhout A. J. Bleecker E. R. Postma D. S. (2009) Identification of PCDH1 as a novel susceptibility gene for bronchial hyperresponsiveness. Am. J. Respir. Crit. Care Med. 180, 929–935 19729670 10.1164/rccm.200810-1621OC PMC2778155

[27] Patterson N. Price A. L. Reich D. (2006) Population structure and eigenanalysis. PLoS Genet. 2, e190 17194218 10.1371/journal.pgen.0020190 PMC1713260

[28] Purcell S. Neale B. Todd-Brown K. Thomas L. Ferreira M. A.R. Bender D. Maller J. Sklar P. de Bakker P. I. W. Dalym M. J. Sham P. C. (2007) PLINK: a toolset for whole-genome association and population-based linkage analysis. Am. J. Hum. Genet. 81, 559–575 17701901 10.1086/519795 PMC1950838

[29] Pruim R. J. Welch R. P. Sanna S. Teslovich T. M. Chines P. S. Gliedt T. P. Boehnke M. Abecasis G. R. Willer C. J. (2010) LocusZoom: regional visualization of genome-wide association scan results. Bioinformatics 26, 2336–2337 20634204 10.1093/bioinformatics/btq419 PMC2935401

[30] Ge D. Zhang K. Need A. C. Martin O. Fellay J. Urban T. J. Telenti A. Goldstein D. B. (2008) WGAViewer: software for genomic annotation of whole genome association studies. Genome Res. 18, 640–643 18256235 10.1101/gr.071571.107 PMC2279251

[31] Team R. C. (2012) R: A Language and Environment for Statistical Computing, R Foundation for Statistical Computing, Vienna

[32] Dixon A. L. Liang L. Moffatt M. F. Chen W. Heath S. Wong K. C. Taylor J. Burnett E. Gut I. Farrall M. Lathrop G. M. Abecasis G. R. Cookson W. O. (2007) A genome-wide association study of global gene expression. Nat. Genet. 39, 1202–1207 17873877 10.1038/ng2109

[33] Ward L. D. Kellis M. (2012) HaploReg: a resource for exploring chromatin states, conservation, and regulatory motif alterations within sets of genetically linked variants. Nucleic Acids Res. 40, D930–D934 22064851 10.1093/nar/gkr917 PMC3245002

[34] Barrett J. C. Fry B. Maller J. Daly M. J. (2005) Haploview: analysis and visualization of LD and haplotype maps. Bioinformatics 21, 263–265 15297300 10.1093/bioinformatics/bth457

[35] Stewart C. E. Sayers I. (2013) Urokinase receptor orchestrates the plasminogen system in airway epithelial cell function. Lung 191, 215–225 23408042 10.1007/s00408-013-9450-z

[36] Kamentsky L. Jones T. R. Fraser A. Bray M. A. Logan D. J. Madden K. L. Ljosa V. Rueden C. Eliceiri K. W. Carpenter A. E. (2011) Improved structure, function and compatibility for CellProfiler: modular high-throughput image analysis software. Bioinformatics 27, 1179–1180 21349861 10.1093/bioinformatics/btr095 PMC3072555

[37] Hao K. Bosse Y. Nickle D. C. Pare P. D. Postma D. S. Laviolette M. Sandford A. Hackett T. L. Daley D. Hogg J. C. Elliott W. M. Couture C. Lamontagne M. Brandsma C. A. van den Berge M. Koppelman G. Reicin A. S. Nicholson D. W. Malkov V. Derry J. M. Suver C. Tsou J. A. Kulkarni A. Zhang C. Vessey R. Opiteck G. J. Curtis S. P. Timens W. Sin D. D. (2012) Lung eQTLs to help reveal the molecular underpinnings of asthma. PLoS Genet. 8, e1003029 23209423 10.1371/journal.pgen.1003029 PMC3510026

[38] Postma D. S. Timens W. (2006) Remodeling in asthma and chronic obstructive pulmonary disease. Proc. Am. Thorac. Soc. 3, 434–439 16799088 10.1513/pats.200601-006AW

[39] Barnes P. J. (2000) Mechanisms in COPD: differences from asthma. Chest 117, 10S–14S 10673467 10.1378/chest.117.2_suppl.10s

[40] Wilhelm O. G. Wilhelm S. Escott G. M. Lutz V. Magdolen V. Schmitt M. Rifkin D. B. Wilson E. L. Graeff H. Brunner G. (1999) Cellular glycosylphosphatidylinositol-specific phospholipase D regulates urokinase receptor shedding and cell surface expression. J. Cell. Physiol. 180, 225–235 10395292 10.1002/(SICI)1097-4652(199908)180:2<225::AID-JCP10>3.0.CO;2-2

[41] Behrendt N. Ploug M. Patthy L. Houen G. Blasi F. Dano K. (1991) The ligand-binding domain of the cell surface receptor for urokinase-type plasminogen activator. J. Biol. Chem. 266, 7842–7847 1850423

[42] Andolfo A. English W. R. Resnati M. Murphy G. Blasi F. Sidenius N. (2002) Metalloproteases cleave the urokinase-type plasminogen activator receptor in the D1-D2 linker region and expose epitopes not present in the intact soluble receptor. Thromb. Haemost. 88, 298–306 12195704

[43] Hoyer-Hansen G. Ploug M. Behrendt N. Ronne E. Dano K. (1997) Cell-surface acceleration of urokinase-catalyzed receptor cleavage. Eur. J. Biochem. 243, 21–26 9030717 10.1111/j.1432-1033.1997.0021a.x

[44] Koolwijk P. Sidenius N. Peters E. Sier C. F. Hanemaaijer R. Blasi F. van Hinsbergh V. W. (2001) Proteolysis of the urokinase-type plasminogen activator receptor by metalloproteinase-12: implication for angiogenesis in fibrin matrices. Blood 97, 3123–3131 11342439 10.1182/blood.v97.10.3123

[45] Beaufort N. Leduc D. Rousselle J. C. Magdolen V. Luther T. Namane A. Chignard M. Pidard D. (2004) Proteolytic regulation of the urokinase receptor/CD87 on monocytic cells by neutrophil elastase and cathepsin G. J. Immunol. 172, 540–549 14688365 10.4049/jimmunol.172.1.540

[46] Newgard C. B. Attie A. D. (2010) Getting biological about the genetics of diabetes. Nat. Med. 16, 388–391 20376050 10.1038/nm0410-388

[47] Fink E. Bhoola K. D. Snyman C. Neth P. Figueroa C. D. (2007) Cellular expression of plasma prekallikrein in human tissues. Biol. Chem. 388, 957–963 17696780 10.1515/BC.2007.104

[48] Lilla J. N. Joshi R. V. Craik C. S. Werb Z. (2009) Active plasma kallikrein localizes to mast cells and regulates epithelial cell apoptosis, adipocyte differentiation, and stromal remodeling during mammary gland involution. J. Biol. Chem. 284, 13792–13803 19297327 10.1074/jbc.M900508200 PMC2679480

[49] Motta G. Rojkjaer R. Hasan A. A. Cines D. B. Schmaier A. H. (1998) High molecular weight kininogen regulates prekallikrein assembly and activation on endothelial cells: a novel mechanism for contact activation. Blood 91, 516–528 9427705

[50] Schmaier A. H. Rojkjaer R. Shariat-Madar Z. (1999) Activation of the plasma kallikrein/kinin system on cells: a revised hypothesis. Thromb. Haemost. 82, 226–233 10605708

[51] Miles L. A. Greengard J. S. Griffin J. H. (1983) A comparison of the abilities of plasma kallikrein, beta-factor XIIa, factor XIa and urokinase to activate plasminogen. Thromb. Res. 29, 407–417 6344314 10.1016/0049-3848(83)90244-x

[52] Beaufort N. Debela M. Creutzburg S. Kellermann J. Bode W. Schmitt M. Pidard D. Magdolen V. (2006) Interplay of human tissue kallikrein 4 (hK4) with the plasminogen activation system: hK4 regulates the structure and functions of the urokinase-type plasminogen activator receptor (uPAR). Biol. Chem. 387, 217–222 16497155 10.1515/BC.2006.029

[53] Verweij N. Mahmud H. Mateo Leach I. de Boer R. A. Brouwers F. P. Yu H. Asselbergs F. W. Struck J. Bakker S. J. Gansevoort R. T. Munroe P. B. Hillege H. L. van Veldhuisen D. J. van Gilst W. H. Sillje H. H. van der Harst P. (2013) Genome-wide association study on plasma levels of midregional-proadrenomedullin and C-terminal-pro-endothelin-1. Hypertension 61, 602–608 23381795 10.1161/HYPERTENSIONAHA.111.203117

[54] Mazzieri R. Blasi F. (2005) The urokinase receptor and the regulation of cell proliferation. Thromb. Haemost. 93, 641–646 15841307 10.1160/TH05-01-0021

[55] Lund L. R. Green K. A. Stoop A. A. Ploug M. Almholt K. Lilla J. Nielsen B. S. Christensen I. J. Craik C. S. Werb Z. Dano K. Romer J. (2006) Plasminogen activation independent of uPA and tPA maintains wound healing in gene-deficient mice. EMBO J. 25, 2686–2697 16763560 10.1038/sj.emboj.7601173 PMC1500865

[56] Davies D. E. (2009) The role of the epithelium in airway remodeling in asthma. Proc. Am. Thorac. Soc. 6, 678–682 20008875 10.1513/pats.200907-067DP PMC2797070

[57] Cohen L. E X. Tarsi J. Ramkumar T. Horiuchi T. K. Cochran R. DeMartino S. Schechtman K. B. Hussain I. Holtzman M. J. Castro M. (2007) Epithelial cell proliferation contributes to airway remodeling in severe asthma. Am. J. Respir. Crit. Care Med. 176, 138–145 17463414 10.1164/rccm.200607-1062OC PMC1994213

[58] Feener E. P. Zhou Q. Fickweiler W. (2013) Role of plasma kallikrein in diabetes and metabolism. Thromb. Haemost. 110, 434–441 23676986 10.1160/TH13-02-0179 PMC3757113

[59] LaRusch G. A. Mahdi F. Shariat-Madar Z. Adams G. Sitrin R. G. Zhang W. M. McCrae K. R. Schmaier A. H. (2010) Factor XII stimulates ERK1/2 and Akt through uPAR, integrins, and the EGFR to initiate angiogenesis. Blood 115, 5111–5120 20228268 10.1182/blood-2009-08-236430 PMC2890145

[60] Wachtfogel Y. T. Kucich U. James H. L. Scott C. F. Schapira M. Zimmerman M. Cohen A. B. Colman R. W. (1983) Human plasma kallikrein releases neutrophil elastase during blood coagulation. J. Clin. Invest. 72, 1672–1677 6556194 10.1172/JCI111126 PMC370455

[61] Marceau F. Regoli D. (2004) Bradykinin receptor ligands: therapeutic perspectives. Nat. Rev. Drug Discov. 3, 845–852 15459675 10.1038/nrd1522

[62] Wilcox-Adelman S. A. Wilkins-Port C. E. McKeown-Longo P. J. (2000) Localization of urokinase type plasminogen activator to focal adhesions requires ligation of vitronectin integrin receptors. Cell. Adhes. Commun. 7, 477–490 11051458 10.3109/15419060009040305

[63] Wei Y. Tang C. H. Kim Y. Robillard L. Zhang F. Kugler M. C. Chapman H. A. (2007) Urokinase receptors are required for alpha 5 beta 1 integrin-mediated signaling in tumor cells. J. Biol. Chem. 282, 3929–3939 17145753 10.1074/jbc.M607989200

