# Supplementary material for: Genome-wide protein QTL mapping identifies human plasma kallikrein as a post-translational regulator of serum uPAR levels
Source: FASEB J. 2014 Feb;28(2):923–34. doi: 10.1096/fj.13-240879 (PMC3898658; doi:10.1096/fj.13-240879)
Supplement: Supplemental Data [file supp_28_2_923__index.html]

Genome-wide protein QTL mapping identifies human plasma kallikrein as a post-translational regulator of serum uPAR levels — Genome-wide protein QTL mapping identifies human plasma kallikrein as a post-translational regulator of serum uPAR levels — Supplemental Data 

# Genome-wide protein QTL mapping identifies human plasma kallikrein as a post-translational regulator of serum uPAR levels

## Supplemental Data

**Files in this Data Supplement:**

- Supplemental Data - (*13-240879SuppData.zip; compressed file 290 KB*)
