# Supplementary material for: Genome-wide protein QTL mapping identifies human plasma kallikrein as a post-translational regulator of serum uPAR levels
Source: FASEB J. 2014 Feb;28(2):923–34. doi: 10.1096/fj.13-240879 (PMC3898658; doi:10.1096/fj.13-240879)
Supplement: Supplemental Data [file supp_fj.13-240879_13-240879SuppData.zip › SF1.pdf]

Query SNP: rs4253238 and variants with r^2 >= 0.8

chr	pos (hg19)	LD (r^2)	LD (D')	variant	Ref	Alt	AFR freq	AMR freq	ASN freq	EUR freq	SiPhy cons	Promoter histone marks	Enhancer histone marks	DNAse	Proteins bound	eQTL tissues	Motifs changed	GENCODE genes	dbSNP func annot
4	187139939	0.97	0.99	rs12331618	A	G	0.66	0.59	0.66	0.52							Pbx-1,Zfp410	KLKB1	
4	187142010	0.81	0.97	rs202221183	TC	T	0.64	0.54	0.62	0.48							17 altered motifs	KLKB1	
4	187142011	0.97	0.99	rs34970894	CA	C	0.66	0.59	0.65	0.52							5 altered motifs	KLKB1	
4	187143802	0.98	0.99	rs11132382	T	G	0.66	0.58	0.66	0.52							7 altered motifs	KLKB1	
4	187145222	0.98	0.99	rs4862669	G	C	0.67	0.59	0.66	0.52								KLKB1	
4	187148133	0.99	1	rs2048	G	T	0.66	0.59	0.66	0.52		HepG2					BCL,BDP1,HNF4	KLKB1	
4	187148387	1	1	rs4253238	C	T	0.80	0.59	0.66	0.52			HepG2				8 altered motifs	KLKB1	
4	187149540	0.99	1	rs1912826	G	A	0.66	0.59	0.66	0.52		NHLF	HepG2, H1	FibroP,Osteobl,HA-sp			4 altered motifs	KLKB1	intronic
4	187150478	0.99	1	rs3775298	A	G	0.80	0.59	0.66	0.52							Hoxc10	KLKB1	intronic
4	187150710	0.98	0.99	rs1511801	A	T	0.79	0.59	0.66	0.52							DMRT2	KLKB1	intronic
4	187152142	0.99	0.99	rs4241815	C	T	0.66	0.59	0.66	0.52							ZEB1	KLKB1	intronic
4	187152327	0.99	0.99	rs4241816	A	T	0.66	0.59	0.66	0.52				Osteobl			13 altered motifs	KLKB1	intronic
4	187152658	0.99	0.99	rs4241817	T	C	0.66	0.59	0.66	0.52								KLKB1	intronic
4	187153786	0.99	0.99	rs4241818	T	C	0.66	0.59	0.66	0.52								KLKB1	intronic
4	187155488	0.99	1	rs4253248	G	A	0.66	0.59	0.66	0.52							5 altered motifs	KLKB1	intronic
4	187156504	0.92	0.99	rs142201367	T	18-mer	0.77	0.57	0.65	0.50							10 altered motifs	KLKB1	intronic
4	187157140	0.81	0.99	rs4241819	C	T	0.60	0.56	0.65	0.47							E2F,Myc,Zfp161	KLKB1	intronic
4	187157458	0.99	0.99	rs4253252	T	G	0.65	0.59	0.66	0.52							CDP,Duxl,Pbx-1	KLKB1	intronic
4	187158034	0.99	0.99	rs3733402	G	A,C	0.79	0.59	0.66	0.52								KLKB1	missense
4	187158508	0.98	0.99	rs4253254	C	T	0.66	0.59	0.66	0.52							Ik-1	KLKB1	intronic
4	187158922	0.99	0.99	rs4253255	G	C	0.65	0.59	0.66	0.52							Zbtb3	KLKB1	intronic
4	187161120	0.86	0.98	rs71640036	G	T	0.63	0.57	0.64	0.49								KLKB1	intronic
4	187161211	0.97	0.98	rs66530140	T	C	0.67	0.59	0.66	0.52								KLKB1	intronic
4	187161501	0.97	0.98	rs35984397	G	A	0.65	0.59	0.66	0.52							DMRT1,Mrg1::Hoxa9,XBP-1	KLKB1	intronic
4	187163612	0.83	0.98	rs4253271	G	T	0.60	0.53	0.62	0.48		H1					6 altered motifs	KLKB1	intronic
4	187163614	0.83	0.98	rs4253272	C	T	0.61	0.54	0.62	0.48		H1					5 altered motifs	KLKB1	intronic
4	187164349	0.95	0.98	rs4253281	G	A	0.80	0.59	0.66	0.52		H1			POL2			KLKB1	intronic
4	187164399	0.96	0.98	rs4253282	C	T	0.68	0.59	0.66	0.52		H1					4 altered motifs	KLKB1	intronic
4	187169167	0.86	0.95	rs1973612	C	T	0.60	0.56	0.65	0.51			H1		POL24H8		6 altered motifs	KLKB1	intronic
4	187174683	0.92	0.96	rs4253311	A	G	0.60	0.57	0.66	0.52							HMG-IY,Pou2f2,Pou5f1	KLKB1	intronic
4	187178783	0.92	0.96	rs200039428	A	ACTT	0.58	0.57	0.65	0.52							Cphx	KLKB1	intronic
4	187178788	0.91	0.96	rs33930717	A	9-mer	0.60	0.57	0.65	0.52								KLKB1	intronic

Supplementary Figure 1: HaploReg analysis of rs4253238. HaploReg analysis on SNP rs4253238 identifies that this KLKB1 5`UTR polymorphism is in linkage disequilibrium ($R^2$) with SNPs in the 5`UTR and KLKB1 gene that cause motif changes to KLKB1 and that are responsible for changes in gene regulatory elements. SNP rs4253238 itself is also responsible for a number of motif changes and regulates an enhancer histone marker. This suggests that the rs4253238 locus may be a marker for changes in KLKB1 expression.
