# Supplementary material for: Genome-wide protein QTL mapping identifies human plasma kallikrein as a post-translational regulator of serum uPAR levels
Source: FASEB J. 2014 Feb;28(2):923–34. doi: 10.1096/fj.13-240879 (PMC3898658; doi:10.1096/fj.13-240879)
Supplement: Supplemental Data [file supp_fj.13-240879_13-240879SuppData.zip › Supplementary Figure 2.pdf]

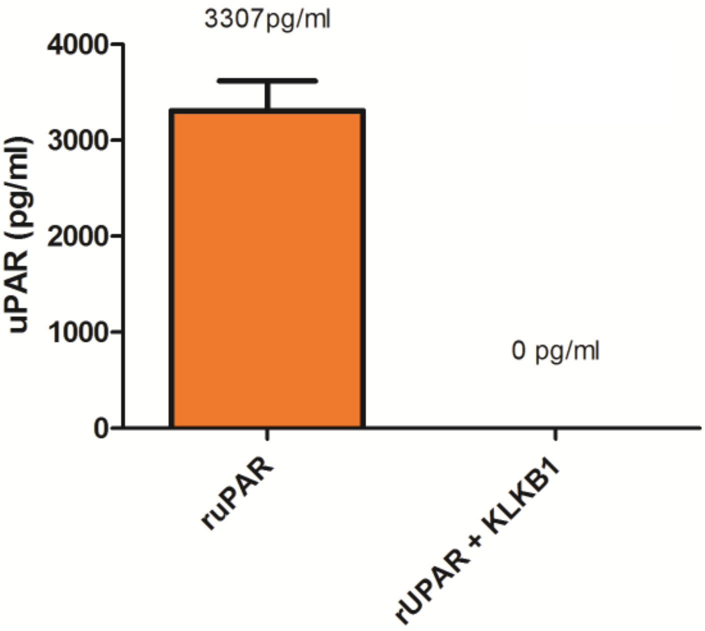

Supplementary Figure 2: KLKB1 digestion of recombinant uPAR (ruPAR) renders it undetectable by ELISA. On incubation with 250ng of KLKB1 at 37°C for twenty-four hours no ruPAR is detectable, identifying that previous measurements of uPAR would likely measure only full length soluble uPAR and not include the KLKB1 digestion fragments.
